# 2,2′-[Nonane-1,9-diylbis(nitrilo­methyl­idyne)]diphenol

**DOI:** 10.1107/S1600536808039858

**Published:** 2008-11-29

**Authors:** Norbani Abdullah, Kong Mun Lo, Hairul Anuar Tajuddin, Jia Ti Tee, Seik Weng Ng

**Affiliations:** a Department of Chemistry, University of Malaya, 50603 Kuala Lumpur, Malaysia

## Abstract

In the title Schiff base compound, C_23_H_30_N_2_O_2_, the complete mol­ecule is generated by crystallographic twofold symmetry, with one C atom lying on the rotation axis. The nonane chain adopts a linear conformation and the hydr­oxy group forms an intra­molecular O—H⋯N hydrogen bond to the imine group.

## Related literature

For the effect of alkyl length on the optical properties of 2,2′-[alkyl-1,9-diylbis(nitrilo­methyl­idyne)]diphenols, see: Kawasaki *et al.* (1996 ▶, 1999 ▶). For the reduction of the Schiff base to the secondary diamine, see: Csaszar (1984 ▶). For the structure of 2,2′-[hexane-1,6-diylbis(nitrilo­methyl­idyne)]diphenol, see: Sheikhshoaie & Sharif (2006 ▶).
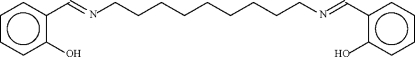


## Experimental

### 

#### Crystal data

C_23_H_30_N_2_O_2_
*M* *_r_* = 366.49
Monoclinic, 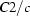

*a* = 43.6905 (10) Å
*b* = 4.7258 (1) Å
*c* = 9.8928 (2) Å
β = 96.935 (2)°
*V* = 2027.65 (8) Å^3^
*Z* = 4
Mo *K*α radiation
μ = 0.08 mm^−1^
*T* = 100 (2) K
0.40 × 0.03 × 0.02 mm

#### Data collection

Bruker SMART APEX CCD diffractometer
Absorption correction: none
8930 measured reflections
2317 independent reflections
1573 reflections with *I* > 2σ(*I*)
*R*_int_ = 0.038

#### Refinement

*R*[*F* ^2^ > 2σ(*F* ^2^)] = 0.040
*wR*(*F* ^2^) = 0.177
*S* = 1.10
2317 reflections
127 parameters
1 restraint
H atoms treated by a mixture of independent and constrained refinement
Δρ_max_ = 0.25 e Å^−3^
Δρ_min_ = −0.23 e Å^−3^

### 

Data collection: *APEX2* (Bruker, 2007 ▶); cell refinement: *SAINT* (Bruker, 2007 ▶); data reduction: *SAINT*; program(s) used to solve structure: *SHELXS97* (Sheldrick, 2008 ▶); program(s) used to refine structure: *SHELXL97* (Sheldrick, 2008 ▶); molecular graphics: *X-SEED* (Barbour, 2001 ▶); software used to prepare material for publication: *publCIF* (Westrip, 2008 ▶).

## Supplementary Material

Crystal structure: contains datablocks global, I. DOI: 10.1107/S1600536808039858/hb2862sup1.cif

Structure factors: contains datablocks I. DOI: 10.1107/S1600536808039858/hb2862Isup2.hkl

Additional supplementary materials:  crystallographic information; 3D view; checkCIF report

## Figures and Tables

**Table 1:** Hydrogen-bond geometry (Å, °)

*D*—H⋯*A*	*D*—H	H⋯*A*	*D*⋯*A*	*D*—H⋯*A*
O1—H1⋯N1	0.86 (1)	1.81 (2)	2.5755 (19)	148 (3)

**Fig. 1.**
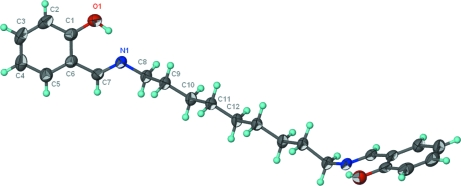
View of the molecular structure of (I) at the 70% probability level. Hydrogen atoms are drawn as spheres of arbitrary radius. The unlabelled atoms are generated by the symmetry operation (1–x, y, 1/2–z).

## supplementary crystallographic information

### Comment

For more details, see the Abstract.  For the molecular structure, see Fig. 1. and for details of hydrogen bonding, see Table 1.

### Experimental

Salicylaldehyde (0.050 mol, 6.1 g) and sodium hydroxide (0.05 mol, 2.0 g) in methanol (125 ml) was added to 1,9-diaminononane (0.025 mol, 3.9 g) in methanol (125 ml). The solution was heated for 1 h. The solvent was evaporated and the product recrystallized from ethanol to yield yellow plates of (I). The rod used for data collection was cut from a plate.

### Refinement

The C-bound hydrogen atoms were placed at calculated positions (C–H = 0.95—0.99 Å) and refined as riding with *U*_iso_(H) = 1.2*U*_eq_(C). The hydroxy H-atom was located in a difference Fourier map and was refined with a distance restraint of O–H = 0.84±0.01 Å.

### Crystal data

**Table:**

C_23_H_30_N_2_O_2_	*F*_000_ = 792
*M**_r_* = 366.49	*D*_x_ = 1.201 Mg m^−^^3^
Monoclinic, *C*2/*c*	Mo *K*α radiation λ = 0.71073 Å
Hall symbol: -C 2yc	Cell parameters from 2058 reflections
*a* = 43.6905 (10) Å	θ = 2.8–27.9º
*b* = 4.7258 (1) Å	µ = 0.08 mm^−^^1^
*c* = 9.8928 (2) Å	*T* = 100 (2) K
β = 96.935 (2)º	Rod, yellow
*V* = 2027.65 (8) Å^3^	0.40 × 0.03 × 0.02 mm
*Z* = 4	

### Data collection

**Table:**

Bruker SMART APEX CCD diffractometer	1573 reflections with *I* > 2σ(*I*)
Radiation source: fine-focus sealed tube	*R*_int_ = 0.038
Monochromator: graphite	θ_max_ = 27.5º
*T* = 100(2) K	θ_min_ = 0.9º
ω scans	*h* = −56→56
Absorption correction: None	*k* = −6→6
8930 measured reflections	*l* = −12→12
2317 independent reflections	

### Refinement

**Table:**

Refinement on *F*^2^	Secondary atom site location: difference Fourier map
Least-squares matrix: full	Hydrogen site location: inferred from neighbouring sites
*R*[*F*^2^ > 2σ(*F*^2^)] = 0.040	H atoms treated by a mixture of independent and constrained refinement
*wR*(*F*^2^) = 0.177	*w* = 1/[σ^2^(*F*_o_^2^) + (0.1065*P*)^2^] where *P* = (*F*_o_^2^ + 2*F*_c_^2^)/3
*S* = 1.10	(Δ/σ)_max_ = 0.001
2317 reflections	Δρ_max_ = 0.25 e Å^−^^3^
127 parameters	Δρ_min_ = −0.23 e Å^−^^3^
1 restraint	Extinction correction: none
Primary atom site location: structure-invariant direct methods	

### Fractional atomic coordinates and isotropic or equivalent isotropic displacement parameters (Å^2^)

**Table:**

	*x*	*y*	*z*	*U*_iso_*/*U*_eq_	Occ. (<1)
O1	0.63065 (3)	0.8920 (3)	0.86876 (12)	0.0364 (4)	
H1	0.6186 (5)	0.787 (5)	0.816 (2)	0.075 (8)*	
N1	0.61425 (3)	0.4953 (3)	0.69663 (13)	0.0283 (4)	
C1	0.66008 (4)	0.8348 (4)	0.84965 (15)	0.0300 (4)	
C2	0.68388 (4)	0.9845 (4)	0.92417 (17)	0.0382 (5)	
H2	0.6793	1.1251	0.9874	0.046*	
C3	0.71405 (4)	0.9284 (4)	0.90596 (19)	0.0410 (5)	
H3	0.7302	1.0303	0.9577	0.049*	
C4	0.72134 (4)	0.7262 (4)	0.81367 (19)	0.0388 (5)	
H4	0.7422	0.6916	0.8009	0.047*	
C5	0.69785 (4)	0.5759 (4)	0.74073 (18)	0.0344 (4)	
H5	0.7027	0.4358	0.6779	0.041*	
C6	0.66712 (4)	0.6252 (3)	0.75711 (15)	0.0281 (4)	
C7	0.64264 (4)	0.4541 (4)	0.68374 (15)	0.0282 (4)	
H7	0.6480	0.3082	0.6249	0.034*	
C8	0.59137 (4)	0.3110 (4)	0.62109 (16)	0.0295 (4)	
H8A	0.5792	0.2140	0.6854	0.035*	
H8B	0.6020	0.1649	0.5720	0.035*	
C9	0.56994 (4)	0.4833 (4)	0.51955 (16)	0.0290 (4)	
H9A	0.5590	0.6250	0.5696	0.035*	
H9B	0.5824	0.5867	0.4584	0.035*	
C10	0.54637 (4)	0.2996 (4)	0.43422 (16)	0.0295 (4)	
H10A	0.5351	0.1845	0.4957	0.035*	
H10B	0.5573	0.1683	0.3786	0.035*	
C11	0.52325 (3)	0.4723 (4)	0.34047 (16)	0.0278 (4)	
H11A	0.5347	0.5929	0.2818	0.033*	
H11B	0.5119	0.5990	0.3968	0.033*	
C12	0.5000	0.2941 (5)	0.2500	0.0291 (5)	
H12A	0.4888	0.1707	0.3082	0.035*	0.50
H12B	0.5112	0.1707	0.1918	0.035*	0.50

### Atomic displacement parameters (Å^2^)

**Table:**

	*U*^11^	*U*^22^	*U*^33^	*U*^12^	*U*^13^	*U*^23^
O1	0.0363 (8)	0.0429 (8)	0.0299 (7)	0.0016 (6)	0.0041 (5)	−0.0045 (5)
N1	0.0256 (8)	0.0366 (8)	0.0213 (7)	0.0014 (6)	−0.0026 (5)	0.0018 (6)
C1	0.0343 (10)	0.0354 (9)	0.0194 (7)	0.0001 (7)	−0.0007 (7)	0.0058 (7)
C2	0.0489 (12)	0.0379 (10)	0.0259 (8)	−0.0070 (8)	−0.0036 (8)	0.0002 (8)
C3	0.0395 (11)	0.0436 (11)	0.0361 (10)	−0.0118 (8)	−0.0115 (8)	0.0069 (8)
C4	0.0286 (10)	0.0441 (11)	0.0415 (10)	−0.0031 (8)	−0.0053 (8)	0.0083 (8)
C5	0.0307 (10)	0.0396 (10)	0.0318 (9)	0.0013 (7)	−0.0006 (7)	0.0039 (7)
C6	0.0298 (9)	0.0331 (9)	0.0204 (7)	0.0006 (7)	−0.0018 (6)	0.0055 (6)
C7	0.0281 (9)	0.0352 (9)	0.0204 (7)	0.0029 (7)	−0.0008 (6)	0.0019 (6)
C8	0.0251 (9)	0.0352 (9)	0.0269 (8)	0.0007 (7)	−0.0017 (7)	0.0005 (7)
C9	0.0238 (9)	0.0354 (9)	0.0266 (8)	0.0014 (7)	−0.0018 (7)	0.0002 (7)
C10	0.0238 (9)	0.0334 (9)	0.0302 (8)	0.0020 (6)	−0.0012 (7)	−0.0005 (7)
C11	0.0231 (8)	0.0332 (9)	0.0265 (8)	0.0013 (6)	0.0005 (6)	−0.0006 (6)
C12	0.0230 (12)	0.0327 (12)	0.0306 (11)	0.000	−0.0010 (9)	0.000

### Geometric parameters (Å, °)

**Table:**

O1—C1	1.349 (2)	C8—C9	1.524 (2)
O1—H1	0.86 (1)	C8—H8A	0.9900
N1—C7	1.277 (2)	C8—H8B	0.9900
N1—C8	1.461 (2)	C9—C10	1.522 (2)
C1—C2	1.393 (2)	C9—H9A	0.9900
C1—C6	1.408 (2)	C9—H9B	0.9900
C2—C3	1.377 (3)	C10—C11	1.523 (2)
C2—H2	0.9500	C10—H10A	0.9900
C3—C4	1.385 (3)	C10—H10B	0.9900
C3—H3	0.9500	C11—C12	1.524 (2)
C4—C5	1.378 (2)	C11—H11A	0.9900
C4—H4	0.9500	C11—H11B	0.9900
C5—C6	1.391 (2)	C12—C11^i^	1.524 (2)
C5—H5	0.9500	C12—H12A	0.9900
C6—C7	1.462 (2)	C12—H12B	0.9900
C7—H7	0.9500		
			
C1—O1—H1	109.1 (19)	C9—C8—H8B	109.6
C7—N1—C8	118.06 (14)	H8A—C8—H8B	108.1
O1—C1—C2	119.15 (16)	C10—C9—C8	112.43 (14)
O1—C1—C6	121.25 (15)	C10—C9—H9A	109.1
C2—C1—C6	119.60 (16)	C8—C9—H9A	109.1
C3—C2—C1	119.86 (17)	C10—C9—H9B	109.1
C3—C2—H2	120.1	C8—C9—H9B	109.1
C1—C2—H2	120.1	H9A—C9—H9B	107.9
C2—C3—C4	121.26 (17)	C9—C10—C11	112.75 (14)
C2—C3—H3	119.4	C9—C10—H10A	109.0
C4—C3—H3	119.4	C11—C10—H10A	109.0
C5—C4—C3	118.99 (18)	C9—C10—H10B	109.0
C5—C4—H4	120.5	C11—C10—H10B	109.0
C3—C4—H4	120.5	H10A—C10—H10B	107.8
C4—C5—C6	121.38 (17)	C10—C11—C12	114.05 (15)
C4—C5—H5	119.3	C10—C11—H11A	108.7
C6—C5—H5	119.3	C12—C11—H11A	108.7
C5—C6—C1	118.89 (15)	C10—C11—H11B	108.7
C5—C6—C7	120.52 (15)	C12—C11—H11B	108.7
C1—C6—C7	120.54 (15)	H11A—C11—H11B	107.6
N1—C7—C6	121.81 (15)	C11—C12—C11^i^	112.9 (2)
N1—C7—H7	119.1	C11—C12—H12A	109.0
C6—C7—H7	119.1	C11^i^—C12—H12A	109.0
N1—C8—C9	110.26 (14)	C11—C12—H12B	109.0
N1—C8—H8A	109.6	C11^i^—C12—H12B	109.0
C9—C8—H8A	109.6	H12A—C12—H12B	107.8
N1—C8—H8B	109.6		
			
O1—C1—C2—C3	179.92 (15)	C2—C1—C6—C7	176.40 (14)
C6—C1—C2—C3	0.6 (2)	C8—N1—C7—C6	−179.01 (14)
C1—C2—C3—C4	0.5 (3)	C5—C6—C7—N1	−179.81 (16)
C2—C3—C4—C5	−1.1 (3)	C1—C6—C7—N1	2.8 (2)
C3—C4—C5—C6	0.6 (3)	C7—N1—C8—C9	−117.30 (16)
C4—C5—C6—C1	0.5 (2)	N1—C8—C9—C10	177.84 (13)
C4—C5—C6—C7	−176.97 (15)	C8—C9—C10—C11	175.42 (14)
O1—C1—C6—C5	179.62 (14)	C9—C10—C11—C12	177.98 (12)
C2—C1—C6—C5	−1.0 (2)	C10—C11—C12—C11^i^	178.76 (15)
O1—C1—C6—C7	−3.0 (2)		

Symmetry codes: (i) −*x*+1, *y*, −*z*+1/2.

### Hydrogen-bond geometry (Å, °)

**Table:**

*D*—H···*A*	*D*—H	H···*A*	*D*···*A*	*D*—H···*A*
O1—H1···N1	0.86 (1)	1.81 (2)	2.5755 (19)	148 (3)
